# Army Correspondence

**Published:** 1862-09

**Authors:** 


### ARMY CORRESPONDENCE.

HUNTSVILLE, ALABAMA, CONVALESCENT BARRACKS.

The mails have been so irregular, that I have refrained from writing heretofore. Having the good fortune to receive a letter of two months age, I write you hoping that you may receive this as soon.

Those who came down sanitarily with amputating cases and desires before Corinth, were doomed, you remember, to cursing and disappointment. I, with previously exalted ideas of Kentucky mammothness, chivalry, hunting and health, have been doomed to the latter only, leaving the cursing to those who can do the subject scientific justice.

The scales have fallen from my eyes, and the comparative microscopy of Kentucky soldiers, while placed in contrast with Northern troops, glaringly appears. The Kentuckians do well in the fight, but in the camp they are worse than useless. They take no care of the camp, of their equipage, or of themselves. I have been connected with one of those regiments, and speak with authority. The testimony of hospital surgeons is, that too great a proportion of Kentucky troops are "hospital pets," and frequent Convalescent Camps and Barracks.

Most of the diseases are chronic diarrhœas and remittent fevers. Both need stimulants. Diarrhœas yield to no local treatment. They must be attacked constitutionally. Whiskey and quinine, with some aromatic and warm spice, is the most successful remedy. How depletants can be used successfully in the army, I see not. A *good article* of whiskey saves more men than any Patent Medicine man ever *advertised* to do, from Townsend's Sarsaparilla to Day & Martin's Blacking. This from me, a thorough going and absolute tee-totaller, is strong language, but I cannot abate from it.

The familiar treatise on Alcohol will not find many converts in the army, and I think that a few months service down here would correct many of the views of "the father of the American Med. Ass." Alcoholic stimulants are absolutely indispensable to maintain life in many, who will return home, and shortly be able to re-enlist.

Remittents do not succumb to the use of quinine so quickly and universally as with us. Larger doses are necessary and often imperative. I rely much on rest, good diet, and a plentiful use externally of cold water, during the accession of the fever. Arsenic does better as an antiperiodic, than with us even; but anything except quinine is difficult to obtain. That, however, is probably owing more to the neglect of the regimental surgeons, than to any defect in the Medical Purveyor's Department.

Scorbutus is prevalent, but in a mild form, showing itself first in sore mouths, which quickly yield to chlorate of potash. "Cold sores" in the mouth become a species of indolent ulcers, but over which nit. arg. exercises no control, excepting to make them worse. If a soldier has had the misfortune to have a broken bone in years previous, the scorbutic state occasions great pain in the neighborhood of the fracture, while at other times the limbs become œdematous and weak, insomuch that they are of no use to the patient.

To scorbutus, I place often the indirect cause of diarrhœa. The men craving vegetable food, on every occasion that presents itself, rush to every excess, including green watermelons and all kinds of young weeds, as "greens." Such trash cannot be imprisoned with impunity in an enfeebled stomach and intestines, and the imprudence results in a stampede on the part of the intestines, and a general "run on the bank," till the offending matter is removed. Soldiers generally cannot be fools; according to the old adage, experience teaches fools: for experience teaches the soldier nothing. Nothing alarmed at the previous course of watermelon treatment; the soldier embraces the very first opportunity to repeat his suicidal action, and soon becomes exhausted beyond remedial aid. It would seem that many of the soldiers in coming to the army, left their quota of common sense at home, relying solely on the force of circumstances, to rejoin it at the end of the war.

Typhoid fever is not so common as I expected to find. "Camp fever" does not decimate our troops as the fearful fogies of editorial-physic tendency, predicted when advocating a progressive "On to Richmond" movement. It is almost always complicated with a scorbutic taint, and after a comparatively short course tends to a typhus rather than a typhoid condition; the tongue is red and raw like a raw steak; the patient unconscious or wild and muttering; the pulse quick, weak, and intermittent. To this state camp fever usually tends, unless met early with stimulants, and par excellence, alcoholic.

Nostalgia is another disease which is not a stranger to us. I had one case whose inception occurred soon after I had discharged 45 men from the regiment at one time. He gradually lost his appetite, lost his spirits and ambition; took no notice of any one, but a surgeon whom he always pestered for a discharge, alleging that he knew he shouldn't get well, although he complained of no pain. At last he became so emaciated, that I sent him to the hospital, where he was carried forth one morning to rest in his long home.

I have had cases of another disease, whose symptoms are as varying as Proteus. It generally occurs just before a battle is expected, and is oftenest seen among the officers, particularly captains and lieutenants; sometimes diarrhœa, or tormina and tenesmus, sometimes like cholera morbus, almost always attacking the alimentary canal somewhere; sometimes the patient has every symptoms of disease you can mention, and a general sense of "feeling awfully." They always recover when the necessity of a battle is over.

The closing of the mail puts an end to these rambling notes.

Yours Respectfully, D.

